# A Cost-Effective Method for Preparing Robust and Conductive Superhydrophobic Coatings Based on Asphalt

**DOI:** 10.1155/2020/5642124

**Published:** 2020-12-24

**Authors:** Wenbin Li, Yong Wang, Yanting Feng, Qing Wang, Xuexia Xu, Guowei Li, Guozhen Dong, Shangqian Jing, Ersong Chen, Xiaoliang Fan, Peng Wang

**Affiliations:** ^1^State Grid Hebei Electric Power Research Institute, Shijiazhuang 050021, China; ^2^State Grid Hebei Energy Technology Service Co., Ltd., Shijiazhuang 050021, China; ^3^School of Energy, Power and Mechanical Engineering, North China Electric Power University, Baoding 071003, China

## Abstract

The wide application of superhydrophobic materials is mainly hindered by the poor mechanical robustness and complicated preparation method. To overcome these problems, we tried to make a combination of hierarchical and self-similar structure by the means of a simple spraying method. By adding nanofiller (carbon nanotube) and microfiller (graphite powder and expanded graphite), the hierarchical structure was constructed. By further doping the fillers in the commercial asphalt uniformly, the self-similar structure was prepared. Based on the aforementioned work, the as-prepared sample could withstand the sandpaper abrasion for 12.00 m under 4.90 kPa. Moreover, this superhydrophobic coating demonstrated good conductivity, superior self-cleaning property, and excellent corrosion resistance. The integration of conductivity with the superhydrophobicity might open new avenues for ground grid applications.

## 1. Introduction

The steel is widely used in our daily life and industry due to its relatively low price, excellent mechanical strength, and superior machinability [1–3]. However, most steel is prone to be corroded, which results in massive economic losses. Many methods have been developed to prohibit the corrosion [4–7]. Particularly, superhydrophobic materials, which can be fabricated by the combination of micro/nanostructure and low surface energy, are attracting more and more attention because the water droplets can maintain nearly spherical shape on them and roll off easily [8–11]. Based on this extreme repellency, many researches have tried to use the superhydrophobic materials to protect corrosion [12–14]. For instance, Cao et al. fabricated a superhydrophobic film which could protect the metal substrate for a long time [15]. Zhang et al. prepared a superhydrophobic coating by combining the epoxy resin with carbon nanotubes, which can effectively protect the Q235 carbon steel [16].

However, the Achilles' heel for superhydrophobic surfaces is the poor mechanical durability. Most superhydrophobic surfaces are prone to be damaged by a slight scratch, or even finger contact [17, 18]. To overcome this weakness, three different methods were developed. First, Lu et al. introduced a “Paint+Adhesive” method which tried bonding the hydrophobic particles using the adhesives [19]. Second, Verho et al. adapted hierarchical structure which tried to utilize the relatively robust microstructure to protect the fragile nanostructure [20]. Third, the self-similar structure is also a potential method which let the new exposed part maintain superhydrophobicity because they are similar in texture and functionality with the abrade parts [21].

The electrical conductivity is also a key consideration because it is crucial in many practical applications. For instance, Q235 steel is widely used as ground grid in the electricity substation due to its cheap price and relatively low electrical resistance. Then, the excellent electrical conductivity should be guaranteed when we tried to use superhydrophobic coating to protect the ground grid [22]. To achieve superhydrophobicity and excellent conductivity simultaneously, scattering carbon-based fillers in the polymer matrix is the main solution. For instance, Hejazi et al. fabricated hair-like superhydrophobic carbon nanotube structure using a template method, which achieved superhydrophobicity without any modification [23]. Gu et al. prepared superhydrophobic surface by coating polystyrene onto the carbon nanotube membrane [24]. Wang et al. constructed a superhydrophobic coating by mixing the graphene with the polydimethylsiloxane [25]. Nevertheless, the aforementioned conductive/superhydrophobic materials have the weakness of mechanical robustness.

In our previous work, a superhydrophobic/conductive material based on the mixture of epoxy and carbon nanotubes has been prepared [26]. The asphalt is widely used in our daily life as the pavement due to its outstanding mechanical strength [27]. Compared with the epoxy, the asphalt has many different specialties, which lead to some special applications such as the pavement. Here, we tried to use the asphalt as the basement to enhance the mechanical strength. In order to obtain self-similar structure, the conductive fillers were uniformly dispersed in the asphalt matrix. In order to further obtain hierarchical structure, both microscale filler (graphite powder and expanded graphite) and nanoscale filler (multiwall carbon nanotube) were utilized. Thus, the hierarchical and self-similar structures were combined which endowed the superhydrophobic coating with outstanding conductivity, excellent mechanical robustness, and superior self-cleaning performance. Moreover, this superhydrophobic coating was achieved by simply spraying, which has the potential for large-scale production.

## 2. Materials and Methods

### 2.1. Materials

The 10# asphalt, 70# asphalt, Q235 steel plate, and expanded graphite (EG) were purchased from a local market. The multiwalled carbon nanotubes with a mean diameter and length of 9.5 nm and 1.5 *μ*m were purchased from Nanocyl Co. Ltd., Belgium (NC7000). 1*H*,1*H*,2*H*,2*H*-per-fluorooctyltriethoxysilane (C_8_F_13_H_4_Si(OCH_2_CH_3_)_3_, FAS) were purchased from Aladdin Reagent Co., Ltd., Shanghai, China. All other chemicals were bought from Sinopharm Chemical Reagent Co., Ltd. (SCRC, China) and used as received.

### 2.2. The Pretreatment of the Q235 Plate

The Q235 steel plates with dimensions of 20 mm × 20 mm × 1.5 mm were utilized as the substrates. Before using, the Q235 substrates were abraded to be the 800# sandpaper and then ultrasonically cleaned in deionized water.

### 2.3. Preparation of the Superhydrophobic Coating

**Figure 1:**
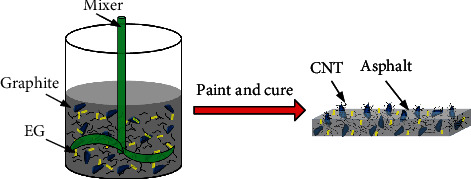
The schematic of the fabrication process.

The schematic of the fabrication process can be found in Figure 1. First, 1.6 g 10# asphalt and 0.4 g 70 # asphalt were mixed into 7.0 g tetrahydrofuran (THF) by mechanical stirring for 4 h, which was marked as solution A. Separately, 0.6 g graphite powder, 0.15 g MWCNTs, 0.03 g EG, and 0.2 g FAS were sequentially dissolved into 8 g THF by means of mechanical stirring, which was defined as solution B. It should be noted that we further altered the amount of MWCNTs and EG for the purpose of improving the electrical conductivity. Then, the solution A was mixed with solution B. After stirring for 4 h, the mixture A was obtained. In the next step, the mixture A was sprayed onto the Q235 steel with the help of spray gun under the pressure of 0.4 MPa. Finally, the coating was cured at room temperature for 24 h. The thickness of the as-prepared coating was ~0.45 mm.

### 2.4. Characterization

The surface microstructures of the superhydrophobic coating were investigated by a scanning electron microscope (SEM, TESCAN Vega3), and the element compositions were assessed from the equipped energy-dispersive spectroscopy (EDS). Before the SEM test, a thin Au film (~2-3 nm) was sputtered onto the samples. The true color confocal microscope was employed to measure the surface roughness. The water contact angles (CAs) were evaluated by a home-made contact angle meter. A high-speed camera (Revealer 2F04) was utilized to assess the sliding angles (SAs). The 5 *μ*L water droplets were adapted in the aforementioned CA and SA tests.

For the resistivity test, the coating was sprayed on a glass slide (76 mm × 26 mm × 2 mm). A DC bridge (QJ84, Shanghai Zhengyang Instrument Factory, China) was utilized to investigate the volume resistivity according to Chinese standard GB/T 2439-2001. The volume resistivity (*ρ*) was calculated as follows:

(1) $$\begin{array}{c} \rho =R\times \frac{S}{L}, \end{array}$$

where *R* is the electrical resistance of the coating, *S* is the cross-sectional area, and *L* is the length of the coating. The conductivity (*s*) is the reciprocal of the volume resistivity.

The CHI760E electrochemical workstation (Shanghai CH Instruments) was utilized to investigate the polarization curves. We used the three-electrode system and set the scanning rate at 1 mV/s. The specimen and a platinum electrode were adapted as the working and counter electrode, respectively. A saturated calomel electrode (SCE) was employed as the reference electrode.

### 2.5. The Abrasion Test

In the abrasion test, the superhydrophobic coating was faced down to the rough surface of 200# SiC sandpaper. Then, a weight of 200 g (4.90 kPa) was put on the top of the Q235 substrate. With the help of external force, the specimen was dragged along the ruler for 20 cm, which was defined as one abrasion cycle.

## 3. Results and Discussion

**Figure 2:**
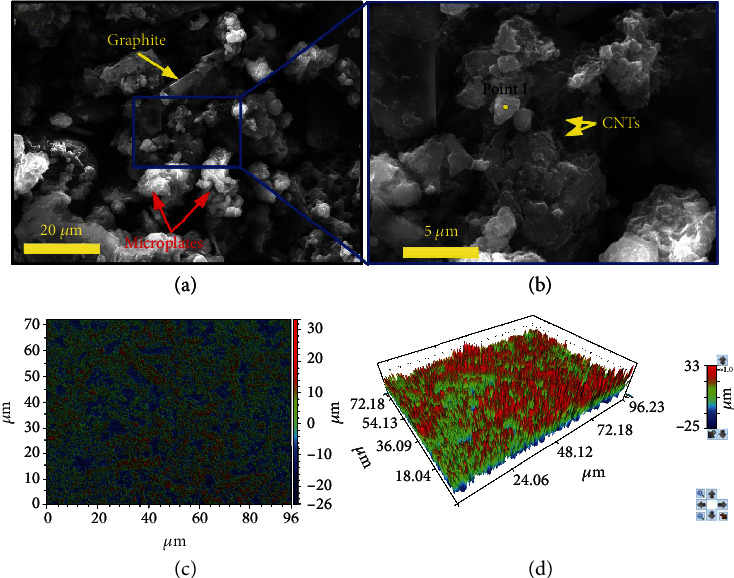
The SEM image of the as-prepared superhydrophobic sample with (a) low and (b) high magnification. (c) 2D and (d) 3D true color confocal microscope images of the as-prepared sample.

In this study, the hierarchical structure was prepared by means of adding both microscale filler and nanoscale filler. The SEM observation confirmed the existence of the hierarchical structure. Many microscale bulges (2-20 *μ*m) could be found from Figure 2(a), which came from the EG and graphite. We further amplified the magnification of SEM observation. Then, many MWCNTs could be found which were on and between the microbulges (Figure 2(b)). We further utilized the true color confocal microscope to evaluate the surface morphology (Figures 2(c) and 2(d)). The surface roughness was calculated to be ~6.55 *μ*m, indicating the microscale roughness of the as-prepared superhydrophobic sample.

**Figure 3:**
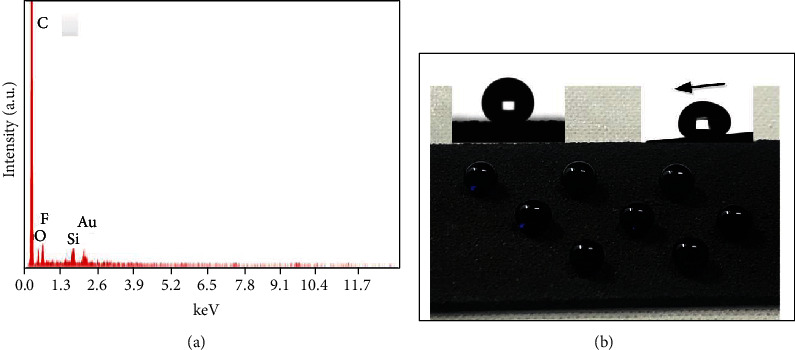
(a) EDS test of point 1 in Figure 2(b). (b) Photograph of water drops deposited on the superhydrophobic sample.

To detect the chemical composition, the EDS measurement was carried out at the area of Figure 2(b). The C, O, F, and Si components could be detected from Figure 3(a). We attributed the F component to the FAS, which played a vital role in the low surface energy. The Au component came from the Au sputtering prior to the SEM observation. Therefore, this coating obtained superhydrophobicity by means of the combination of hierarchical structure and the low surface energy. It can be found that eight spherical shape water droplets were randomly scattered on the surface (Figure 3(b)), indicating the outstanding superhydrophobicity. We further calculated the CAs and SAs to quantitatively evaluate the wetting state, and this coating exhibited a high CA of 163° and a low SA of 5°. Thus, it is reasonable to deduce that this coating demonstrated stable Cassie-Baxter state where the water droplets were suspended on the surface [28, 29].

**Figure 4:**
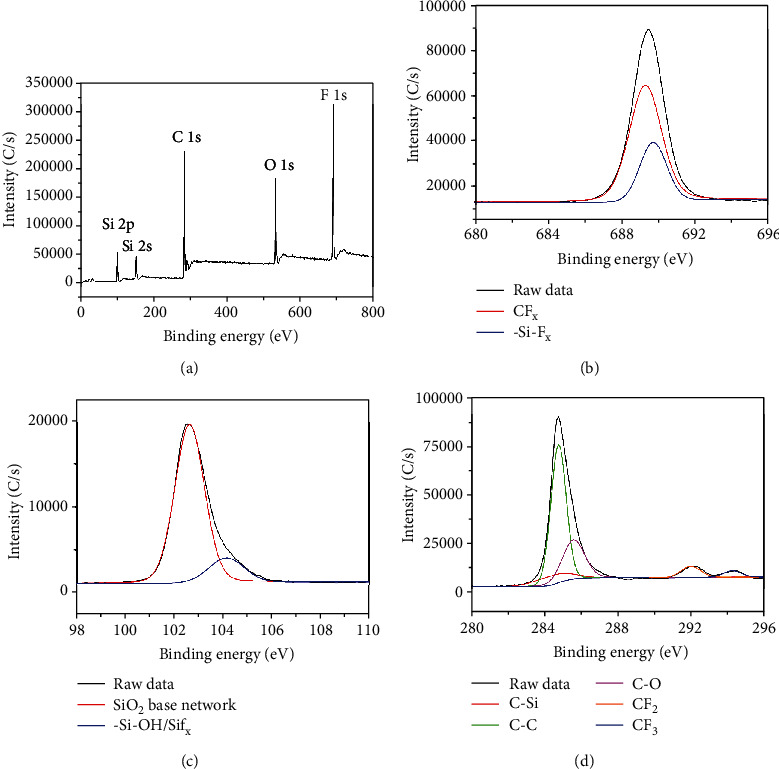
XPS measurement of the graphene superhydrophobic composite. (a) Survey XPS spectrum of the as-prepared superhydrophobic surface. (b) F 1s, (c) Si 2p, and (d) C 1s XPS spectrum of the sample.

XPS measurement was performed to further investigate the surface chemical composition of the as-prepared superhydrophobic surface.  Figure 4 shows the survey spectra of the sample. It can be found that the Si 2p, Si 2s, C 1s, O 1s, and F 1s peaks are detected from the surface.  Figure 4(b) shows curve-fitted F 1s core-level spectra of the sample. A dominant peak appearing at 689.32 eV corresponds to fluorine bonded as CF_*x*_ in the FAS chain indicating that F is present in same bonding environment as that of FAS [30]. A shoulder peak at higher binding energy of 689.75 eV could be associated with Si-F_*x*_ interaction [31]. This confirms the presence of fluoride groups on the silica particles [32]. Curve-fitted Si 2p core-level spectra of the sample are showed in Figure 4(c). Si 2p core-level spectra show a dominant peak at 104.15 eV, which corresponds to -Si-OH or Si-F_*x*_ species. Low-intense component peak around 102.64 eV could be due to SiO_2_-based network.  Figure 4(d) shows the multielement spectra of C 1s; observed peaks at 284.50, 284.78, 285.60, 292.00, and 294.27 eV are ascribed to C-Si, C-C, C-O, CF_2_, and CF_3_, respectively [30–32].

The universal utilization of superhydrophobic materials is hindered by their poor robustness. In other words, the micro/nanostructures which are indispensable for constructing the superhydrophobicity are destructed easily. To solve this problem, the asphalt was utilized as the binder which is widely used in pavement for its excellent mechanical strength and bonding force. At the same time, both micro- and nanofillers were doped in the asphalt matrix to guarantee the formation of hierarchical structure which would enhance the mechanical durability. The sandpaper abrasion test has been widely utilized to evaluate the mechanical robustness [33–41]. Tian et al. further pointed out that the abrasion distance and applied pressure are two key factors to facilitate comparison among different researches [42].

**Figure 5:**
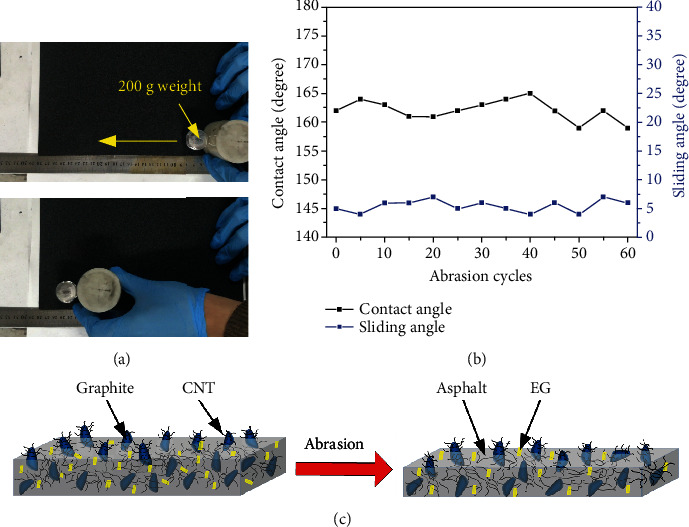
(a) Images of the processes and results of destruction by sandpaper abrasion. (b) The CAs and SAs on the as-prepared sample as a function of abrasion cycle. (c) The schematic of antiabrasion mechanism.

Thus, the sandpaper abrasion test was performed in this research. First, the sample was faced down to the rough surface of SiC sandpaper (200#). Then, a weight of 200 g (4.90 kPa) was put on the top of the sample, as shown in Figure 5(a) and Movie S1. By applying the external force, the sample moved along the ruler for 20 cm as one abrasion cycle. Although some powders could be found during the abrasion process, the superhydrophobicity was retained by watching the behavior of dropping waters. The quantitative changes in CAs and SAs during the abrasion process could be further found in Figure 5(b). Even after 60 abrasion cycles, the CA was 159° and the SA was 6°, indicating the outstanding mechanical robustness. Meanwhile, it was found that the thickness of the coating reduced from ~0.45 mm to ~0.10 mm. We ascribe the retention of superhydrophobicity to the self-similar structure (Figure 5(c)), which makes the undamaged layer of the superhydrophobic coating similar to the exposed parts in functionality and texture.

The conductivity of the materials has received more and more attention recently. On the one hand, the conductive coating could shed off the accumulated electrons and then improve the reliability of the microelectronic device. On the other hand, the excellent conductivity is indispensable for some special application such as the ground grid. In this research, we tried to optimize the electrical property by means of altering the amount of the MWCNTs and EGs. The electrical conductivity and antiabrasion ability were investigated simultaneously, and the results are summarized in Table 1. The detailed test method of the electrical conductivity and antiabrasion ability can be found in the experiment section. With the addition of conductive fillers, the electrical conductivity is increased, but the antiabrasion property decreased simultaneously. Thus, how to make a balance between the electrical conductivity and antiabrasion property is a crucial challenge. After optimization, this coating could exhibit a conductivity of 42.46 S/m, which could maintain superhydrophobicity after 60 abrasion cycles.

**Figure 6:**
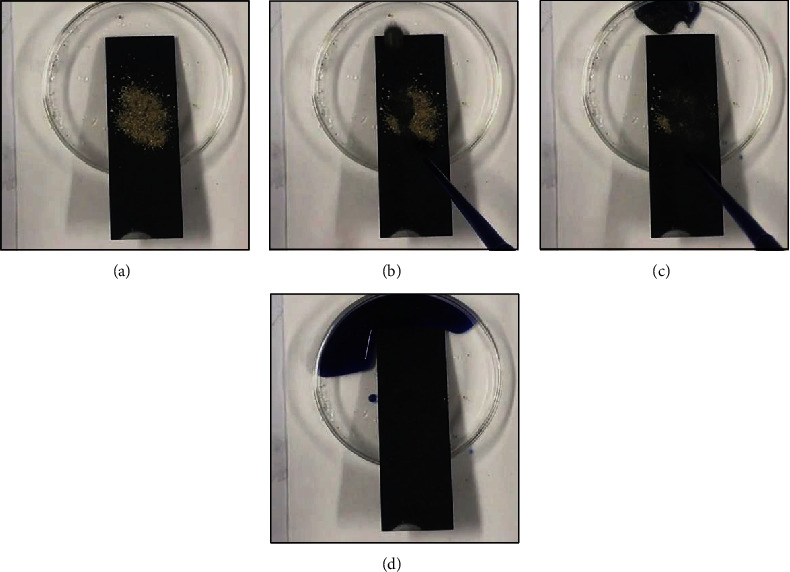
Self-cleaning process on the as-prepared superhydrophobic surface: (a) the surface contaminated by the soil powder; (b, c) the contaminated surface dropped with water droplets; (d) the contaminated surface after the water dropping process.

Here, the soil was utilized as the model contaminant, and the glass slide was used as the substrate. As shown in Figure 6(a) and Movie S2, we first placed the superhydrophobic sample at a slope angle of ~15° and then spread a layer of soil onto the sample. In the next step, the water droplets were dribbled onto the sample. It can be found that the water droplets rolled down easily (Figures 6(b) and 6(c)). Once the water droplet contacted the soil particles, it would take the contaminant array due to the extremely low water adhesion. Finally, a completely clean sample was obtained (Figure 6(d)), suggesting the excellent self-cleaning property.

**Figure 7:**
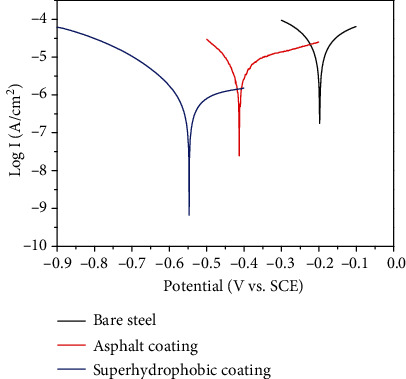
Tafel polarization curves of bare Q235 steel, epoxy coating, and superhydrophobic coating.

Moreover, this superhydrophobic coating exhibited excellent corrosion resistance [43–45]. The steel is universally utilized in our daily life due to their excellent mechanical strength, easy processing, and cost efficiency. Corrosion is regarded as the main failure mechanism for steel (especially for Q235 steel). In this research, we tried to improve the anticorrosive performance by means of superhydrophobic coating. The polarization curves were utilized to evaluate the corrosion resistance, as shown in Figure 7. Here, we employed the 3.5 wt% NaCl aqueous solutions as the electrolyte. Before the electrochemical test, we immersed the samples into the electrolyte for 3 h. Because the corrosion potential (*E*_corr_ vs. SCE) and the corrosion current density (*i*_corr_) are two crucial for assessing the anticorrosive performance, we further deduced them from the polarization curves. The corrosion potentials for the bare Q235 steel, Q235 steel with epoxy coating, and superhydrophobic coating were calculated to be -0.198, -0.413, and -0.547 v, respectively. Furthermore, the corrosion current density for the bare Q235 steel, Q235 steel with epoxy coating, and superhydrophobic coating was calculated to be 2.41 × 10^−5^, 6.24 × 10^−6^, and 6.91 × 10^−7^ A/cm^2^, respectively. Thus, the superhydrophobic coating could reduce the corrosion current density 35 folds compared with the bare sample and reduce 9 folds compared with the epoxy coating. We ascribed the outstanding corrosion resistance to superhydrophobicity. When the superhydrophobic coating was immersed in a corrosive solution, the micro/nanostructures of the coating tend to trap the air, which will serve as a cushion and hold back the electron transfer between Q235 steel substrate and the corrosive electrolyte [46, 47].

## 4. Conclusion

In summary, we reported a simple and cost-effective method to prepare superhydrophobic/conductive asphalt. The conductivity of the asphalt can be controlled by adding conductive filler. When the filler was dispersed uniformly in the asphalt matrix, the self-similar structure can be obtained. Moreover, the hierarchical structure could be constructed by adding nanoscale filler (carbon nanotube) and microscale filler (graphite powder and expanded graphite). Due to the combination of self-similar and hierarchical structure, this superhydrophobic asphalt demonstrated mechanical robustness to the sandpaper abrasion. Moreover, this asphalt composite exhibited good conductivity, superior self-cleaning property, and excellent corrosion resistance.

## Figures and Tables

**Table 1:** The experiment results of the superhydrophobicity with different contents of the filler.

Experiment	MWCNT (g)	EG (g)	Conductivity (S/m)	Antiabrasion (cycles)
1	0.10	0.00	1.96	600
2	0.15	0.03	4.26	300
3	0.20	0.04	8.53	80
4	0.30	0.05	42.46	60
5	0.35	0.06	138.14	5

## Data Availability

The video data used to support the findings of this study are included within the supplementary information files.
