# Supplementary figures and images for: A Cost-Effective Method for Preparing Robust and Conductive Superhydrophobic Coatings Based on Asphalt
Source: Scanning. 2020 Dec 24;2020:5642124. doi: 10.1155/2020/5642124 (PMC7775183; doi:10.1155/2020/5642124)

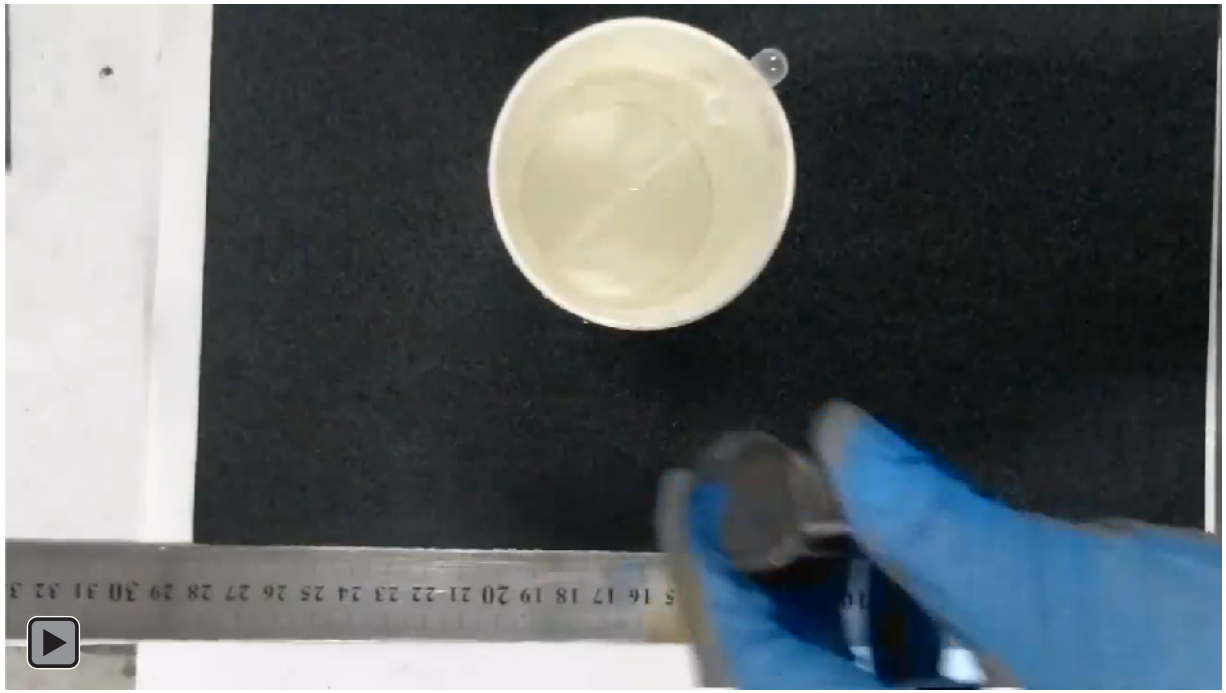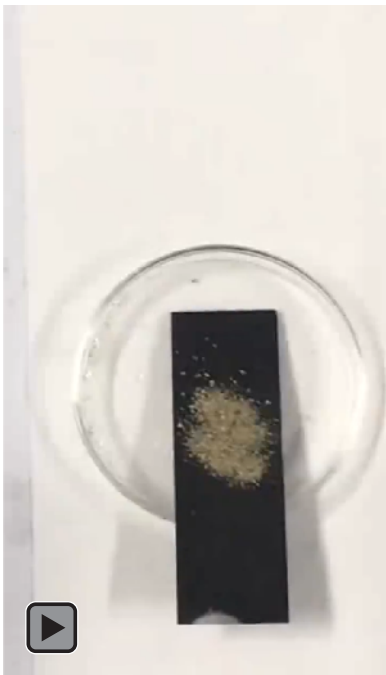

Supplement: Supplementary Materials — The following are available online. Movie S1: the antiabrasion test. Movie S2: the self-cleaning test. [file 5642124.f1.pdf]
